# Psychosocial characteristics influence the duration of hospitalization in patients with psychotic disorders

**DOI:** 10.1192/j.eurpsy.2021.1431

**Published:** 2021-08-13

**Authors:** A. Velelekou, E. Tsomaka, K. Tsaras, D. Papagiannis, I. Papathanasiou, E. Fradelos

**Affiliations:** 1 Ag. Markela, Psychiatric Hospital of Attica, Aigaleo, Greece; 2 Psychiatric Department, Sotiria general hospital, Athens, Greece; 3 Nursing Department, University of Thessaly, Larissa, Greece; 4 General, University of Thessaly, Larissa, Greece

**Keywords:** Duration of hospitalization, psychosis, schizophrénia

## Abstract

**Introduction:**

Schizophrenia spectrum disorders are related with prolonged stay in hospital and high cost for treating them. As a consequence, the determination of the factors that affect the duration of hospitalization is essential.

**Objectives:**

The purpose of the study is the determination of the psychosocial characteristics of inpatients in a public psychiatric hospital and their association with the duration of hospitalization.

**Methods:**

A total of 103 patients with a diagnosis of Schizophrenia (according to ICD-10) participated in the study. The socio demographic characteristics were recorded and the following psychometric tools were used: NEO- Five Factor Inventory, Connor-Davidson Resilience Scale (CD-RISC25), Multidimensional Scale of Perceived Social Support, Multidimensional Scale of Perceived Social Support (MSPSS), Positive and Negative Syndrome Scale (PANSS), Global Assessment of Functioning scale (GAF).All instruments were adapted to greek population. All statistical analysis was performed using SPSS v.25.

**Results:**

The median length of hospital stay was 40,7 days. The number of previous admissions (p=0,010), the type of admission (compulsory or voluntary) (p=0,017), the physical restrain (p=0,043), the duration of restrain (p=0,002) as well as the existence of social support networks and in particular social support from friends (p=0,018), seem to affect the duration of hospitalization.

**Conclusions:**

The present study underlines the signification of the psychosocial factors that could contribute to the prediction of longer hospitals stays, the planning of appropriate interventions and as a result the reduction of hospital costs.

